# Supplementary figures and images for: Managing flowering time in Miscanthus and sugarcane to facilitate intra- and intergeneric crosses
Source: PLoS One. 2021 Jan 7;16(1):e0240390. doi: 10.1371/journal.pone.0240390 (PMC7790387; doi:10.1371/journal.pone.0240390)

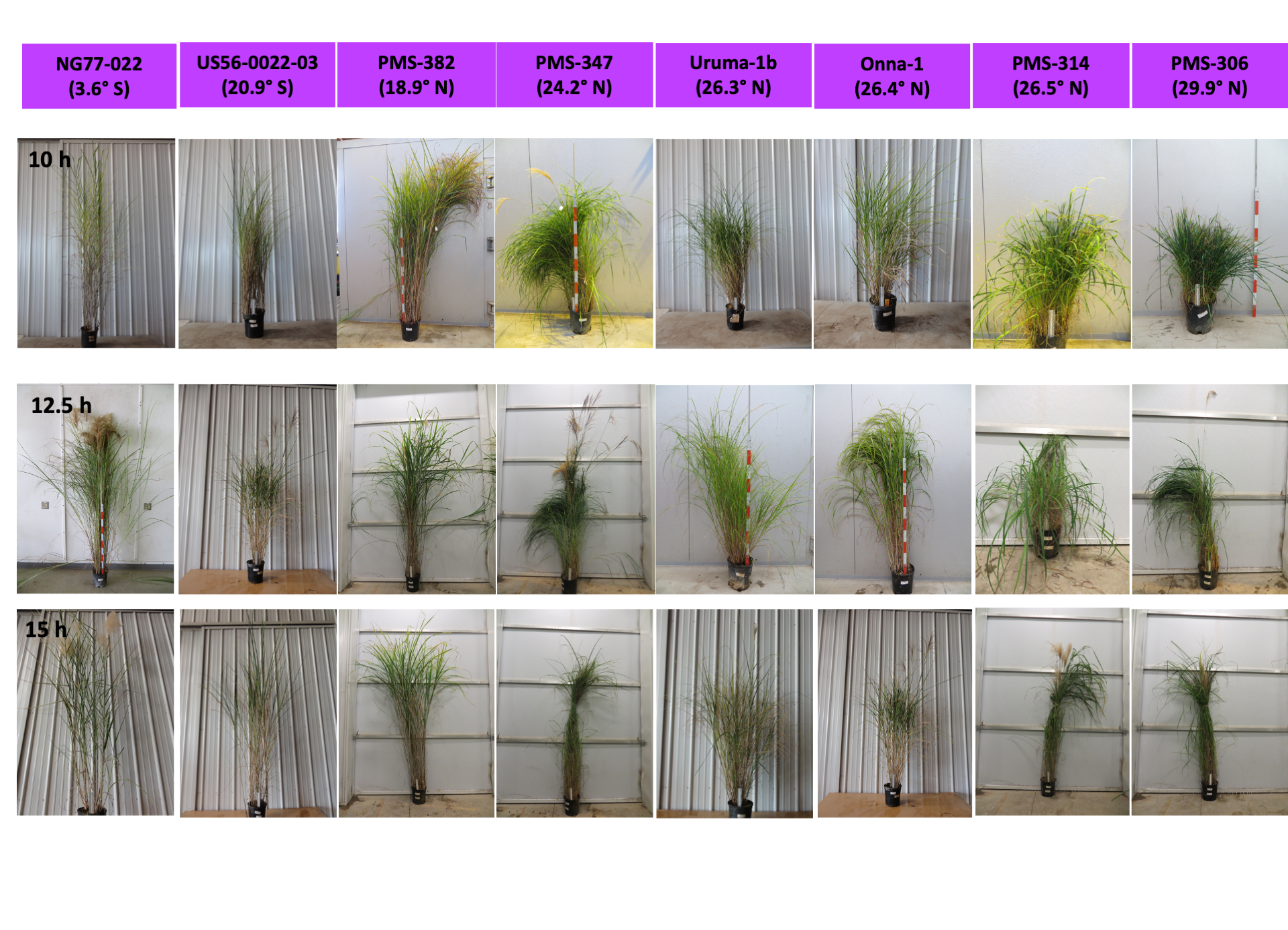

Supplement: S1 Fig — Plants were tested under each of three day lengths: 10, 12.5, and 15 h. Colored background behind Miscanthus genotype names represent the M. sinensis genetic groups identified by Clark et al. [23, 24], which included Korea/North China (red), Yangtze-Qinling (green), Northern Japan (blue), Southern Japan (yellow), Sichuan Basin (orange), and Southeastern China plus tropical (purple). In each photo, plant size is scaled by either a 20 cm ruler (black and white) or a 1 m stick (orange and white). (TIF) [file pone.0240390.s001.tif]

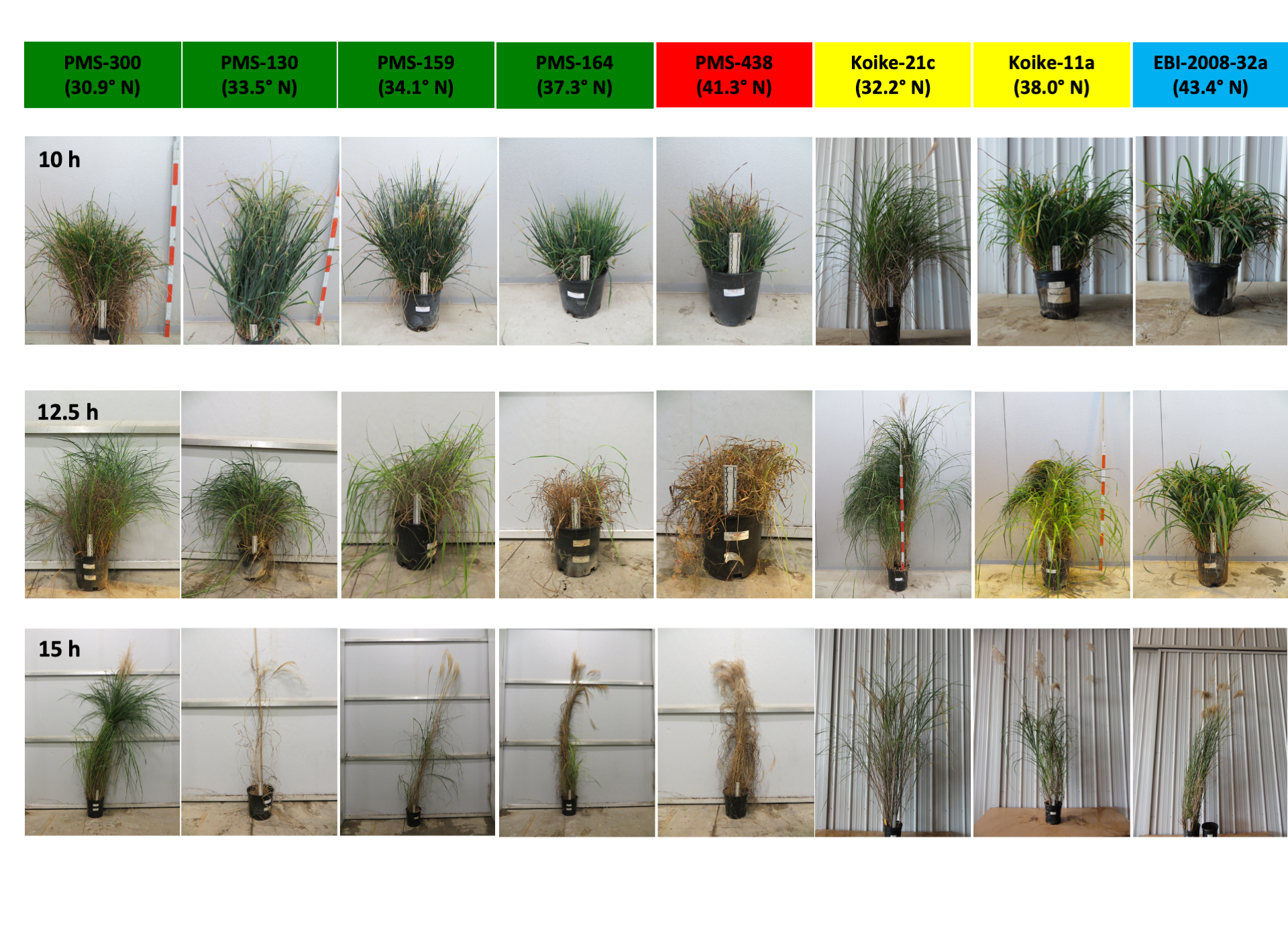

Supplement: S2 Fig — Plants were tested under each of three day lengths: 10, 12.5, and 15 h. Colored background behind Miscanthus genotype names represent the M. sinensis genetic groups identified by Clark et al. [23, 24], which included Korea/North China (red), Yangtze-Qinling (green), Northern Japan (blue), Southern Japan (yellow), Sichuan Basin (orange), and Southeastern China plus tropical (purple); for interspecific hybrids (PMS-300) between M. sacchariflorus and M. sinensis, the dominant M. sinensis genetic group is shown. In each photo, plant size is scaled by either a 20 cm ruler (black and white) or a 1 m stick (orange and white). Note that accessions originating from high latitudes typically remained short and had few or no flowering stems when grown under short days but were taller and flowered when grown under long days. (TIF) [file pone.0240390.s002.tif]
