# Supplementary material for: Managing flowering time in Miscanthus and sugarcane to facilitate intra- and intergeneric crosses
Source: PLoS One. 2021 Jan 7;16(1):e0240390. doi: 10.1371/journal.pone.0240390 (PMC7790387; doi:10.1371/journal.pone.0240390)
Supplement: Attachment — Submitted filename: Response to Reviewers.docx [file pone.0240390.s009.docx]

We greatly appreciate the comments from two reviewers and the editor. We have made revisions based on reviewer’s comments. We replaced the original Figs 3, 4, and 5 with new figures. The geographic background of original Figs 3-5 was made using ArcGIS. For the new Figs 3-5, we made the geographic background using the R package ggmap (Kahle & Wickham, 2013), which is a free, non-copyrighted, open-source programming package. I wrote my own R codes to develop these three new figures. Therefore, no copyright related issues should exist. I have also made my R codes used in this study publicly available on GitHub (<https://github.com/hxdong-genetics/Geographic-map-in-Miscanthus-flowering-study>).

Reference

Kahle D, Wickman H. ggmap: Spatial visualization with ggplot2. 2013. The R Journal. 5: 144-161

Reviewer #1: In this manuscript entitled " Manage flowering time in Miscanthus and sugarcane to facilitate intra- and intergeneric crosses” to synchronize flowering time of Saccharum and Miscanthus species by using different cultural treatments. They found the barriers to synchronizing the flowering of sugarcane and Miscanthus, and also proposed methods. For Miscanthus, flowering dates may be obtained by staggered plantings grown under a single conducive constant day length, or by planting on a single date and growing under conducive and constant day lengths. The important application of the article is used to improve breeding program of crops by controlling flowering time of sugarcane and Miscanthus. In general, this paper is technically accurate, and interesting. I suggest the authors do some minor revisions, before accepting.
We appreciate the reviewer’s meticulous review on our manuscript. We have made minor revisions based on reviewer’s comments.

Minor comments
1. Lin 276-277 The first flowering flush was observed from October 2014 to January 2015, with… Please recheck, October 2014 to Dec 2014?

We incorporated the reviewer’s comment.

2. L307 from “5” August 2015 to “19” December 2015 in supplementary table.

We incorporated the reviewer’s comment.

3. L434 to 30 or 60 sec d-1

We double checked the references, previous studies used declining day length ranging from 30 sec to 60 sec per day, not just 30 sec or 60 sec per day. Therefore, we need to keep our original writing.

4. It is redundant in discussion paragraph, I suggest that authors reduce the length.

We think that it is important to keep the current discussion intact. Our study consists of multiple experiments with multiple years. It would be vital to appropriately repeat key information in discussion so that readers could keep track the findings of each experiment.

Reviewer #2: The topic is interesting. Terminology and methods they used in this study are clearly described and acceptable in a scientific journal. The authors present an interesting publication that reports the usefulness of hybridization based on manage flowering time in Miscanthus and sugarcane. The aim of this manuscript is interesting and important for the hybridization based on manage flowering time in Miscanthus and sugarcane. I don’t have any negative recommendations on this manuscript and I will suggest accepting this manuscript in the current form.

We appreciate the reviewer’s kind comments.
